# Comparative Quasi-Static Compressive Loading Performance of Two Ultrathin Ceramic Occlusal Veneers for Minimally Invasive Restorations

**DOI:** 10.3390/biomimetics11070517

**Published:** 2026-07-22

**Authors:** Francisco Garcia-Torres, Juan Pablo Flores-Ortega, Gabriela A. Gamundi-Cantu, Silvia Rojas-Rueda, Jose L. Ayala-Herrera, Mark Adam Antal, Carlos A. Jurado, Hamid Nurrohman

**Affiliations:** 1 Department of Prosthodontics and Implantology, School of Dentistry, University of La Salle Bajio, Leon 37150, Mexico; 2 School of Dental Medicine, Ponce Health Sciences University, Ponce 00716, Puerto Rico; 3 Department of Operative and Esthetic Dentistry, Faculty of Dentistry, University of Szeged, 6720 Szeged, Hungary; 4 Department of Restorative Dentistry & Prosthodontics, The University of Texas School of Dentistry, Houston, TX 77054, USA

**Keywords:** minimally invasive dental restoration, ceramic occlusal veneer, zirconia, lithium disilicate

## Abstract

Background: In the field of minimally invasive restorative dentistry, ultrathin (<0.5 mm thickness) ceramic occlusal veneers are increasing being used as alternatives to full-coverage crowns, particularly for mild occlusal wear and not deep caries. However, only limited research attention has been given to how marked reduction in the thickness of a veneer restoration affects its mechanical performance. The purpose of the present in vitro study was to compare the performance of restorations that comprised a 0.3 mm thick zirconia veneer to the case when a lithium disilicate veneer was used, under quasi-static compressive loading. Methods: Forty extracted human molars, without caries, cracks or fractures and with intact coronal structure, were randomly assigned to two groups: lithium disilicate occlusal veneers (*n* = 20) and zirconia occlusal veneers (*n* = 20). The teeth were embedded in acrylic resin up to the cementoenamel junction. Digital scans were used to record the original anatomy and guide restoration design. Standardized occlusal preparations were performed using a 0.3 mm reduction protocol and verified with silicone guides to support a biomimetic, tooth-preserving approach. After preparation, the teeth were rescanned, and restorations were designed and fabricated using CAD/CAM technology. Lithium disilicate restorations were milled from Ivoclar Porcelain System [IPS], esthetic maximized [e.max] computer-aided design [CAD] blocks, whereas zirconia restorations were milled from Prettau 3 zirconia discs. Restorations were adhesively cemented with dual-cure resin cement following material-specific surface treatment protocols. Fracture resistance was tested using a universal testing machine under compressive loading until failure. Results: The fracture loads with lithium disilicate and zirconia occlusal veneers were 481.45 ± 68.23 N and 720.93 ± 95.44 N, respectively. Fracture was catastrophic in lithium disilicate occlusal veneers whereas it was not so when zirconia veneer was used. Conclusion: Quasi-static compressive fracture load when a lithium disilicate veneer was used was significantly lower than when a zirconia veneer was used.

## 1. Introduction

Biomimetic dentistry follows conservative and minimally invasive principles by preserving natural tooth structure and restoring its biomechanical behavior. It emphasizes early caries detection, remineralization, disease control, and minimal tooth removal [1,2]. Because enamel and dentin are more valuable than restorative materials, only the structure necessary to restore function, esthetics, and integrity should be removed. Adhesive materials and bonding techniques further support this approach by enabling direct bonding to enamel and dentin without extensive mechanical retention [3,4].

Posterior occlusal veneers are a minimally invasive alternative to full-coverage dental crowns [5,6]. These veneers are particularly useful for the management of worn, eroded, or structurally compromised posterior teeth when occlusal coverage is required but complete circumferential preparation can be avoided [7,8,9,10,11,12,13,14,15,16,17,18,19]. Two widely used materials for fabricating these veneers are lithium disilicate and zirconia. Each has its attractive features and shortcomings. Lithium disilicate has a high refractive index (hence, it is esthetically pleasing) and, when etched with hydrofluoric acid and treated with silane, it has high bond strength to dental adhesives, but it also has lower fracture toughness than other ceramics, and it has technique-sensitive cementation for clinicians because it requires carefully treatment prior to cementation. Zirconia has high flexural strength, high fracture toughness and a low crack propagation rate, but it has aesthetic limitations because its high opacity limits the translucency and optical properties, and it is difficult to modify or cut off due to its high strength [20,21].

The appropriate thickness of a restoration is influenced by many variables, notably, the strength of the supporting tooth, the amount of available enamel, the restoration design, and the restoration–tooth bonding protocol [22,23]. Although much work has been reported on this issue when the restoration is a crown [22], this is not the case when it is a veneer restoration. As ceramic thickness decreases, the restoration may become more susceptible to fracture under functional or parafunctional loading, particularly in posterior areas where occlusal forces are greater. Thinner restorations may have reduced bulk to resist tensile stresses, crack initiation, and crack propagation during loading. However, the magnitude of this effect depends on multiple factors, including the type of ceramic material, the quality of the adhesive interface, the restoration design, the elastic modulus of the supporting tooth structure, the cementation protocol, and the presence or absence of enamel for bonding. Moreover, clinical case reports have successfully demonstrated the application of occlusal veneers in posterior dentition [24,25,26,27,28,29,30,31].

These findings are important to establish the minimum acceptable veneer thickness that should be used without compromising the mechanical performance of the veneer under normal occlusal loading conditions. One way of doing so is to compare the resistance, under compressive loading, and morphologies of ultrathin (herein, defined as <0.5 mm thick) veneers fabricated from different materials. Only a very limited amount of work in this area has been reported in the literature. The majority of the studies have been performed on plastic teeth and without following the natural bonding protocols as performed on natural dentition.

The purpose of the present work was to compare the quasi-static compressive fracture load and fracture morphology of 0.3 mm thick lithium disilicate and zirconia posterior occlusal veneers when cemented on human molar teeth.

## 2. Materials and Methods

The study was approved by the University of La Salle Bajío in León, Mexico, as part of a Master’s thesis in the Specialty Program in Prosthodontics.

### 2.1. Specimen Collection

Forty extracted human maxillary third molars were collected from the university dental clinic after extraction for orthodontic or surgical reasons. Male and female patients ranged from age 18 to 45 years old in healthy conditions. Only teeth with intact coronal structures were included. The inclusion criteria were human molars without carious lesions, previous restorations, occlusal wear, or loss of coronal integrity. Teeth were excluded if they presented occlusal fractures, non-carious cervical lesions, enamel hypoplasia, enamel hypomineralization, or any other structural defect.

After collection, all teeth were cleaned with 3% hydrogen peroxide and stored in 0.7% thymol solution.

### 2.2. Specimen Preparation

**Figure 1:**
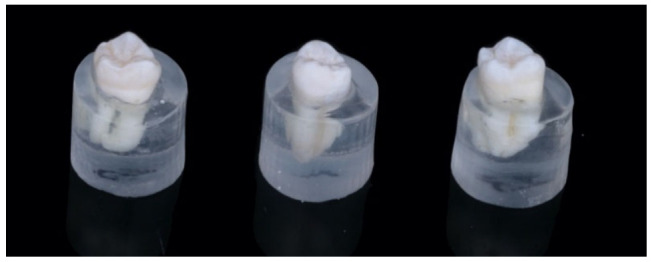
Extracted teeth embedded in transparent acrylic.

Each tooth was embedded in transparent acrylic resin (NicTone, MDC Dental, Guadalajara, Mexico) up to the cementoenamel junction, leaving the coronal portion exposed for scanning, tooth preparation, restoration fabrication, cementation, and mechanical testing (Figure 1).

**Figure 2:**
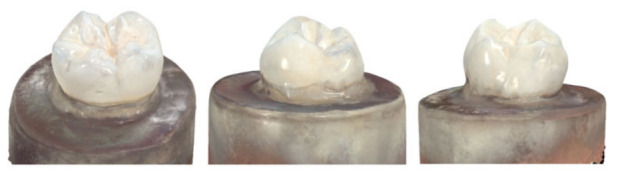
Initial scan of the occlusal surface of the teeth.

Each specimen was initially scanned using an intraoral scanner (3Shape Trios, Copenhagen, Denmark) to obtain a digital file of the original occlusal anatomy. These scans served as a reference to preserve and reproduce the original occlusal morphology during the digital design of the restorations (Figure 2).

**Figure 3:**
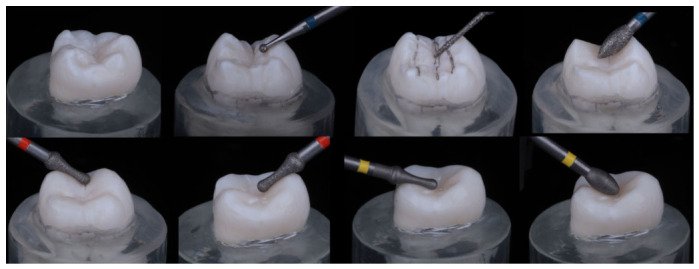
Sequence for tooth preparation with diamond burs.

**Figure 4:**
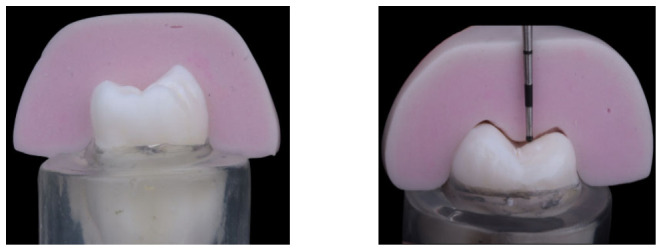
Tooth reduction guides help clinicians achieve the desired amount of tooth preparation.

Silicone reduction guides were fabricated for each tooth using condensation silicone material (Zetalabor, Zhermack, Badia Polesine, Italy). Tooth preparations were performed with diamond burs (M-I-R-A Kit, Jota AG, Rüthi, Switzerland) (Figure 3), and the reduction guides were used throughout the preparation process to ensure a standardized anatomical reduction of 0.3 mm (Figure 4).

**Figure 5:**
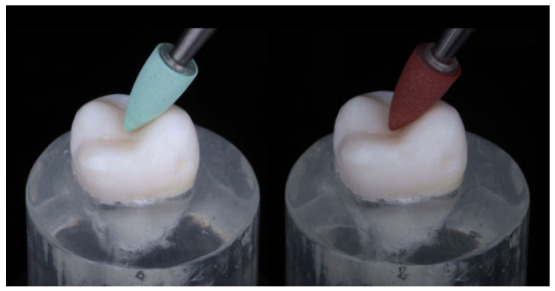
Polishing of the final tooth preparation in order to obtain a smooth surface.

The final preparations were polished using (Ceramic Polishers, Jota AG, Rüthi, Switzerland) to obtain a smooth and standardized surface (Figure 5).

**Figure 6:**
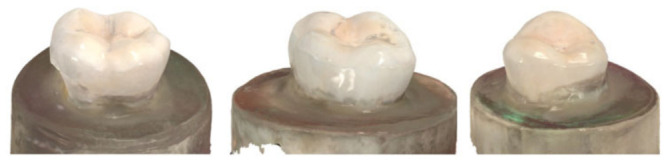
Digital scans of the prepared teeth.

Following polishing of the tooth preparations, each specimen was scanned using a digital scanner (3Shape Trios, Copenhagen, Denmark) (Figure 6). The resulting digital files were used to fabricate the restorations according to the original occlusal anatomy captured in the initial scan.

**Figure 7:**
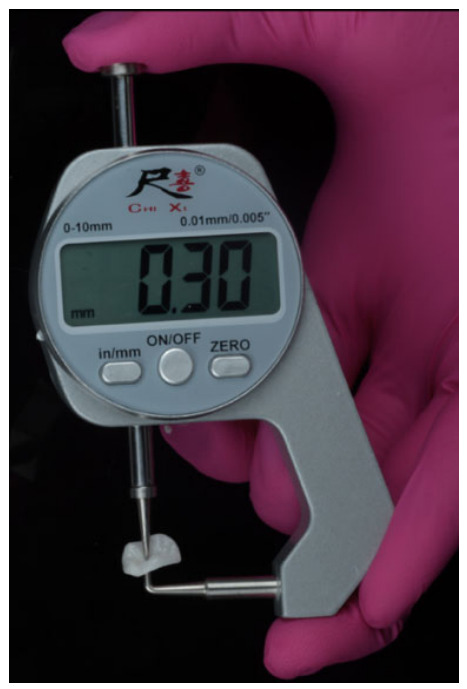
Confirming the 0.3 mm thickness of the ultrathin zirconia occlusal veneer.

Following digital scanning, the restorations were designed using Modellier (Zirkonzahn, Gais, Italy). A total of twenty zirconia restorations (Prettau 3, Zirkonzahn, Gais, Italy) and twenty lithium disilicate restorations (IPS e.max CAD, Ivoclar, Schaan, Liechtenstein) were fabricated. The thickness of each restoration was verified with a digital caliper and standardized at 0.3 mm (Figure 7).

The lithium disilicate restorations were crystallized in a furnace (Programat CS6, Ivoclar, Schaan, Liechtenstein) following the recommended time and temperature, that is, closing time at 403 °C in 6 min, first heating rate 60 °C per minute up to 710 °C, second heating rate bringing the temperature from 840 °C to 850 °C in 1 min, and closing at slow mode to prevent thermal stresses. The zirconia veneers were sintered in a furnace (Programat CS6, Ivoclar, Schaan, Liechtenstein) according to the manufacturers’ instructions in a conventional mode at the recommended time and temperature, that is, heating up rate 8 °C/minute, holding time 2 h, cooling rate 8 °C/minute and final sintering temperature 1600 °C.

**Figure 8:**
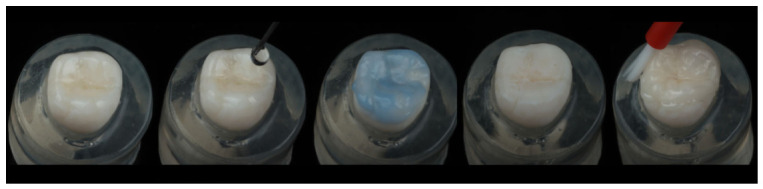
Tooth treatment including airborne-particle abrasion, cleaning with chlorhexidine, application of phosphoric acid, air-drying and primer application.

The tooth-surface treatment for the cementation protocol consisted of airborne-particle abrasion with 20 µm aluminum oxide at 2 bar pressure for 10 s (AquaCare, Velopex International, London, UK), followed by surface cleaning with chlorhexidine. Phosphoric acid gel (Total Etch, Ivoclar, Schaan, Liechtenstein) was then applied to the occlusal surface for 30 s, rinsed thoroughly, and gently air-dried. Primer (Tooth Primer Panavia V5, Noritake Dental, Tokyo, Japan) was subsequently applied, and the restorations were cemented with resin cement (Panavia V5, Noritake Dental, Tokyo, Japan) (Figure 8).

**Figure 9:**
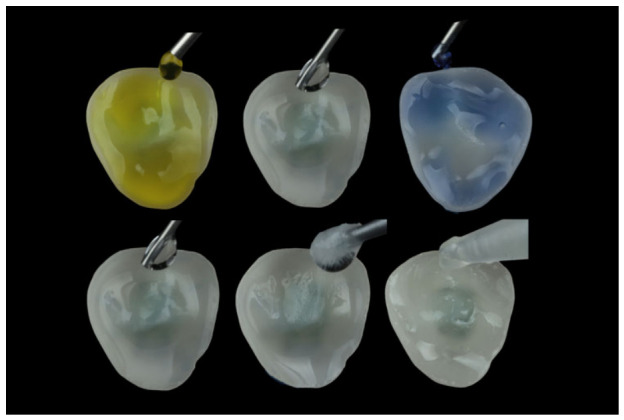
Lithium disilicate treatment with hydrofluoric acid; rinsing and air-drying; phosphoric acid; rinsing and air-drying; primer and cement application.

For the lithium disilicate restorations, ceramic surface treatment began with the application of hydrofluoric acid (Porcelain Etch, Ultradent, South Jordan, UT, USA) for 20 s, followed by rinsing and air-drying. Phosphoric acid (Ultra-Etch, Ultradent, South Jordan, UT, USA) was then applied for 20 s to remove ceramic salt residues and surface contaminants. The restorations were subsequently placed in an ultrasonic bath with alcohol for 5 min to remove any remaining contaminants and acid residues. After ultrasonic cleaning, the restorations were air-dried, and ceramic primer (Clearfil Ceramic Primer Plus, Kuraray Dental, Tokyo, Japan) was applied for 60 s. Finally, resin cement (Panavia V5, Kuraray Dental, Tokyo, Japan) was applied to the restoration and tooth surfaces, and the restorations were cemented according to the manufacturer’s instructions (Figure 9).

**Figure 10:**
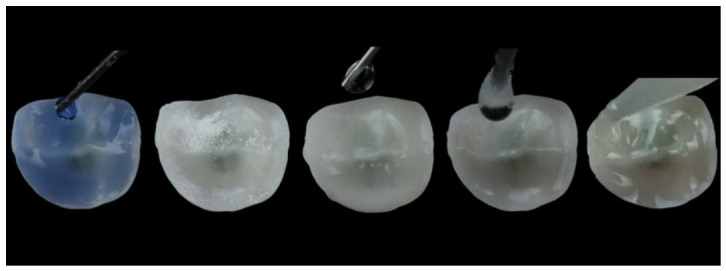
Zirconia treatment: first with cleaning paste, then airborne-particle abrasion; rinsing and air-drying; then primer application; and finally cement application.

For the zirconia restorations, ceramic surface treatment began with cleaning using a zirconia-cleaning paste (ZirClean, Bisco, Schaumburg, IL, USA). The restorations were then treated with airborne-particle abrasion, rinsed, and air-dried. Primer (Clearfil Ceramic Primer Plus, Kuraray Dental, Tokyo, Japan) was applied for 60 s. Finally, resin cement (Panavia V5, Kuraray Dental, Tokyo, Japan) was applied to the restoration and tooth surfaces, and the restorations were cemented according to the manufacturer’s instructions (Figure 10).

Excess resin cement was removed with a microbrush, and light curing was performed using a curing light (Valo, Ultradent, South Jordan, UT, USA) for 20 s on each of the following surfaces: occlusal, mesial, distal, buccal, and lingual.

After cementation, the restorations were polished (Gloss Chairside Set, Jota AG, Rüthi, Switzerland) to obtain a smooth, highly finished surface on the occlusal and marginal areas.

### 2.3. Compressive Test

**Figure 11:**
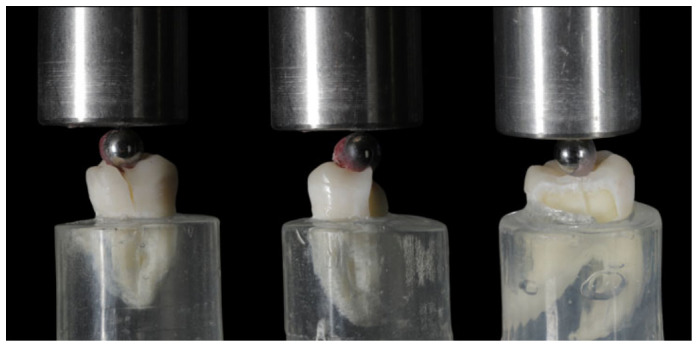
Fracture testing with load application until fracture.

Each restoration was subjected to 10,000 thermal cycles between 5 °C and 55 °C, with a dwell time of 20 s. After that, the restoration was fixed in a jig and subjected to compressive loading until fracture using a universal testing machine (Proline Universal Testing Machine, Zwick Roell, Ulm, Germany) at a crosshead speed of 1.0 mm/min (Figure 11), until fractured. The load was applied with a spherical-shaped indenter positioned at the central area of the occlusal surface. The load required to produce a complete fracture was recorded.

### 2.4. Fractographic Analysis

Fractographic analysis of the fractured specimens was performed using standardized photographic images of each ultrathin occlusal veneer cemented to a natural tooth. The number and lengths of cracks were quantified and compared across the experimental groups.

### 2.5. Statistical Analysis

The Levene test was used to check for homogeneity of variance between the two populations (fracture loads in the lithium disilicate veneer group and the zirconia veneer group). If that was case, the significance of the difference in the means of the two populations was determined using the one-way analysis of variance (ANOVA) test. Significant difference was denoted when *p* < 0.05. All the tests were conducted using a commercially available statistical software package (SPSS v30.0; IBM Corp., NY, USA).

## 3. Results

### 3.1. Compressive Fracture Loads

Homogeneity of variance of the populations of fracture loads in the two study groups was established. The fracture load of the lithium disilicate restorations (481.45 ± 68.23 N) was lower than that of zirconia restorations (720.93 ± 95.44 N) (F(1,38) = 83.323; *p* < 0.001) (Table 1).

### 3.2. Fractographic Analysis

**Figure 12:**
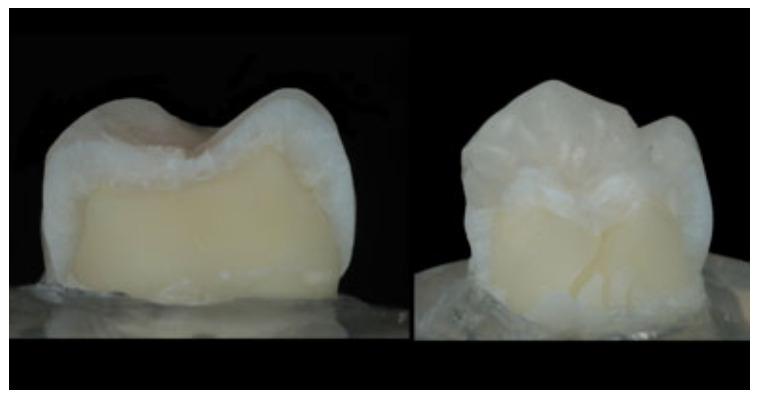
Lithium disilicate fractured specimens.

**Figure 13:**
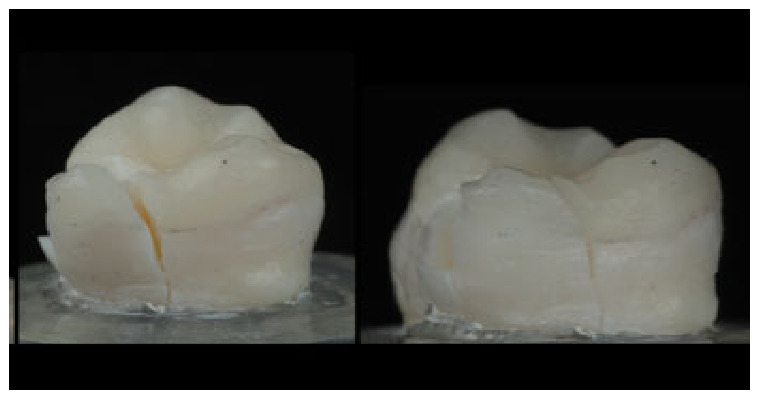
Zirconia fractured specimens.

Lithium disilicate restorations suffered catastrophic failure, characterized by complete detachment of the veneer and, in some specimens, of the underlying tooth (Figure 12). In contrast, zirconia restorations presented visible crack lines but did not experience catastrophic failure or loss of tooth structure (Figure 13).

## 4. Discussion

The purpose of this in vitro study was to compare the fracture resistance of 0.3 mm ultrathin occlusal veneers fabricated from zirconia and lithium disilicate ceramics. These restorations have gained attention as minimally invasive treatment options for posterior teeth because they preserve sound tooth structure; however, reducing ceramic thickness may compromise their mechanical performance under functional loading. Current evidence on the fracture resistance of zirconia and lithium disilicate occlusal veneers remains limited. In addition, many studies have used printed teeth rather than natural teeth and have not followed bonding protocols that closely replicate clinical procedures [32,33,34,35,36]. Therefore, further research using natural teeth and clinically relevant adhesive protocols is needed to provide more realistic and reliable evidence.

Based on the results, lithium disilicate veneers displayed fracture resistance of 481.45 N, which was significantly lower than the value of zirconia veneers at 720.93 N. This difference may be attributed to zirconia’s superior mechanical properties, including higher flexural strength, fracture toughness, and resistance to crack propagation. In contrast, lithium disilicate offers excellent esthetics and predictable adhesive bonding but generally has lower fracture toughness and flexural strength. Therefore, under the conditions tested, zirconia may provide greater mechanical resistance than lithium disilicate when used at an ultrathin thickness of 0.3 mm, which is clinically relevant because the success of these restorations depends on ceramic strength, adhesive bonding, preparation design, and occlusal loading conditions.

The fracture resistance values obtained in this study were 481.45 N for lithium disilicate and 720.93 N for zirconia. Both values exceeded previously reported swallowing forces of approximately 100 N and maximum bite forces of 320 N [37], as well as masticatory forces recorded during the consumption of rye bread, raw carrots, boiled meat, raw cabbage, and cooked meat [38,39,40,41,42]. The significantly higher fracture resistance of zirconia is also consistent with previous studies comparing zirconia and lithium disilicate restorations [43,44]. This difference may be attributed to zirconia’s dense polycrystalline structure, transformation-toughening mechanism, and ferroelastic domain switching, which limit crack propagation [45,46]. In contrast, although lithium disilicate crystals can deflect cracks, its glassy matrix is more susceptible to crack initiation and propagation [47,48].

From a clinical perspective, both materials demonstrated fracture resistance values that may exceed conventional masticatory forces, suggesting that 0.3 mm ultrathin occlusal veneers could represent a conservative restorative option when appropriate case selection, adhesive protocols, and occlusal adjustments are employed. The specimens underwent 10,000 thermal cycles, a protocol reported to simulate approximately 1 year of clinical function and commonly used before mechanical testing of ceramic restorations. Zirconia may be particularly advantageous when restorative space is limited or mechanical demands are high. Failure patterns also differed between materials: lithium disilicate exhibited more catastrophic fractures, including restoration detachment and occasional involvement of the underlying tooth structure, whereas zirconia primarily developed visible cracks without comparable structural damage. Nevertheless, these findings should be interpreted cautiously because clinical loading involves repeated multidirectional forces, moisture, pH variations, thermal changes, and parafunctional habits that cannot be fully reproduced by a single-load in vitro test.

This study has several limitations. Only one veneer thickness, 0.3 mm, and two CAD/CAM ceramic materials were evaluated; therefore, future studies should assess additional thicknesses and other ceramic or ceramic-like materials. Furthermore, thermal cycling alone does not reproduce the combined effects of mechanical fatigue, moisture, salivary enzymes, pH changes, and variations in loading direction. Fracture patterns were assessed visually using photographs, as reported in previous studies [49,50], but scanning electron microscopy would provide a more detailed analysis of failure mechanisms. The teeth were also embedded in acrylic without a periodontal ligament-simulating material. Future investigations should incorporate thermomechanical aging, cyclic fatigue, periodontal ligament simulation, different preparation and cementation protocols, and clinically relevant outcomes such as marginal adaptation, wear, surface roughness, and adhesive degradation. Ultimately, clinical studies are required to determine the long-term survival and complication rates of 0.3 mm zirconia and lithium disilicate occlusal veneers.

## 5. Conclusions

Under quasi-static compressive loading, restorations comprising a zirconia veneer fractured at 720.93 ± 95.44 N and the fractures were not catastrophic (that is, no loss of tooth structure). In contrast, for restorations that comprised lithium disilicate veneer, the fracture load was 481.45 ± 68.23 N and the fractures were catastrophic.

## Figures and Tables

**Table 1:** Fracture resistance value of ultrathin occlusal veneers.

Type of Ceramic	Force at Crack, N (Mean ± SD)	Minimum (N)	Maximum (N)
Lithium Disilicate	481.45 ± 68.23	353.98	577.84
Zirconia Prettau 3	720.93 ± 95.44	541.99	867.59

## Data Availability

All source data may be obtained from the corresponding author.
